# Editorial: Gastrointestinal autonomic disorders

**DOI:** 10.3389/fneur.2024.1492409

**Published:** 2024-09-30

**Authors:** Francisco Tustumi, Vincent Ho, Sophie Clementine Payne, Rafael Bernhart Carra

**Affiliations:** ^1^Department of Gastroenterology, Instituto do cancer do Estado de São Paulo, São Paulo, SP, Brazil; ^2^Department of Health Sciences, Hospital Israelita Albert Einstein, São Paulo, SP, Brazil; ^3^Department of Gastroenterology, Western Sydney University, Penrith, NSW, Australia; ^4^Department of Medical Bionics, University of Melbourne, Melbourne, VIC, Australia; ^5^Bionics Institute, Melbourne, VIC, Australia; ^6^Department of Neurology, Universidade de São Paulo, São Paulo, SP, Brazil

**Keywords:** gastrointestinal autonomic dysfunction, dysmotility, autonomic nervous system, achalasia, gut-brain axis, autonomic denervation

The intricate relationship between gastrointestinal (GI) and nervous systems has been a focal point of research, leading to an increased understanding of how disruptions in this relationship can manifest in various GI disorders (1). This Frontiers Research Topic on Gastrointestinal Autonomic Disorders presents a collection of studies that address GI function, autonomic regulation, and the gut-brain axis. The insights gained from these studies are crucial for understanding the pathophysiology of these disorders and for exploring potential therapeutic interventions.

**Figure 1:**
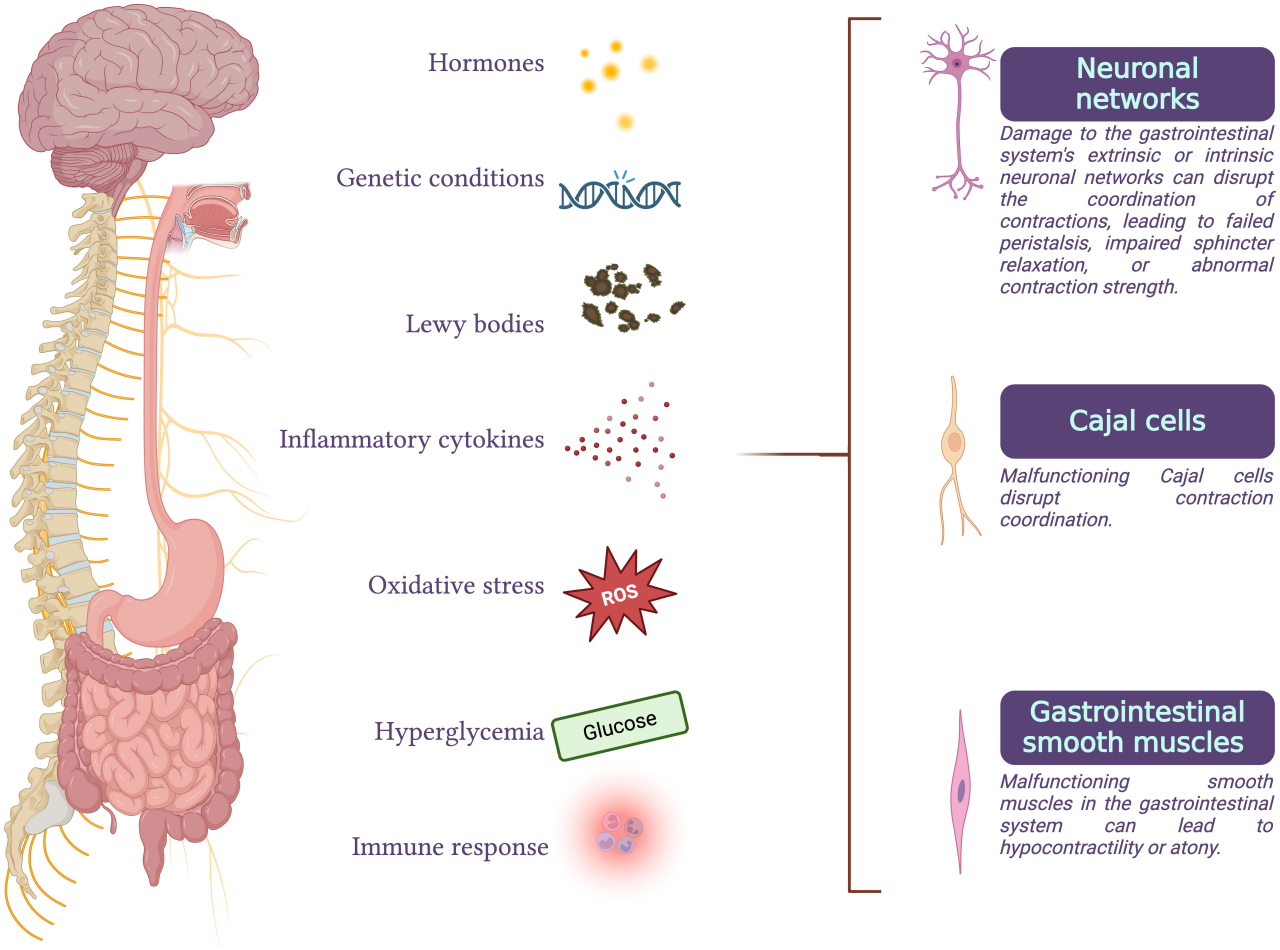
Gastrointestinal functional disorders can arise when there is an impairment in any of the processes of neuronal activity, coordination of peristalsis, or smooth muscle contractility. These disorders can result from degeneration of parasympathetic or sympathetic efferents, malfunction of the enteric nervous system or interstitial cells of Cajal, abnormalities in gastrointestinal smooth muscle cells, or disruptions in the interactions between these processes. Conditions such as hormonal imbalances, genetic disorders, Parkinson's disease, inflammatory or immune responses, oxidative stress, and chronic hyperglycemia are some of the factors that can compromise normal gastrointestinal function.

GI functional disorders can occur when neuronal activity, peristalsis coordination, or smooth muscle contractility are impaired (2) (Figure 1).

GI motility and secretion are regulated by two neuronal networks (2): the extrinsic network, which includes the sympathetic and parasympathetic nervous systems, and the intrinsic network— the enteric nervous system (ENS). The ENS consists of the myenteric (Auerbach's) plexus and the submucosal (Meissner's) plexus. The extrinsic network works in conjunction with the ENS and the central nervous system (3).

Gastroparesis is a complex neuromuscular condition characterized by impaired gastric function with delayed emptying in the absence of mechanical obstruction, resulting in symptoms such as abdominal fullness, and vomiting (4). The most frequent form of gastroparesis, diabetic gastroparesis (DGP), is caused by vagus nerve damage from oxidative stress and inflammatory changes related to chronic high blood sugar levels (5). Additionally, high glucose levels can directly affect the smooth muscle cells of the stomach and can alter the production of hormones and neurotransmitters involved in gastric motility. This combination of neural, muscular, and biochemical disturbances underpins the pathogenesis of DGP. The treatment of DGP is challenging, prompting ongoing efforts to develop new treatment strategies. Gastric electrical stimulation significantly improves patient symptoms (6). Endoscopic procedures such as gastric peroral endoscopic pyloromyotomy (G-POEM) are initially superior to gastric electrical stimulation. However, G-POEM and electrical stimulation have a significant risk for recurrence (7). Vagus nerve stimulation is emerging as a promising treatment for DGP (8), but there remains a lack of well-supported evidence from studies in humans. The potential of alternative therapies is explored in a study that provides an overview of systematic reviews on acupuncture for DGP (Li et al.). Although acupuncture has been suggested as a treatment for DGP, this study critically evaluates the quality of existing reviews. Although pooled studies agree that acupuncture can be effective and has minimal side effects, the overall low quality of the evidence demands careful interpretation.

In addition to diabetes, other less frequent conditions can also lead to gastroparesis; these include idiopathic, post-viral, or conditions related to other neurologic disorders, such as Parkinson's disease, in which Lewy pathology can affect visceromotor fibers (4, 9). Wu and Ho reviewed possible links between gastroparesis and autonomic dysfunction in Ehlers-Danlos syndrome (EDS), a group of inherited disorders that affect connective tissues, which is associated with postural orthostatic tachycardia syndrome (POTS). The review highlights underrecognized links between these conditions, emphasizing the role of autonomic dysfunction in the pathophysiology of GI symptoms in patients with EDS and POTS.

A study published in this Research Topic examines the prokinetic effect of erythromycin in managing gastroparesis in critically ill patients (Szczupak et al.). Critically ill patients are affected by numerous biochemical and hormonal disturbances, such as systemic inflammation and hyperglycemia, ultimately leading to GI smooth cell function impairment and neuronal dysfunctionality (10). Erythromycin, a macrolide antibiotic, enhances gastric motility by acting on smooth muscle receptors in the stomach and myenteric neurons. The authors presented their experience with prokinetic medications for treating critical gastroparesis in ICU patients. In most of their patients, the inclusion of erythromycin led to the resolution of symptoms.

Numerous conditions can impair GI smooth muscle. Chang et al. investigated smooth muscle contractile responses to bile acids in the mouse ileum. The findings revealed that bile acids influence smooth muscle contractility through TGR5 signaling, independent of the sex of the host or the sodium-dependent bile acid transporter ASBT. These findings might guide future targeted therapies for disorders related to altered bile acid homeostasis and GI smooth muscle dysfunction.

Achalasia, a rare motility disorder of the esophagus, can be secondary to immune-related neuronal degeneration of the myenteric plexus, leading to dyscoordination of esophageal contraction and lower esophageal sphincter function (11). In addition, inherited forms of Cajal cell dysfunction can also lead to achalasia (11, 12). The interstitial cells of Cajal transmit signals to the smooth muscle cells, functioning as the stomach's pacemakers. Achalasia significantly impacts patients' eating behaviors. Through a qualitative approach involving focus groups, Kalantari et al. developed a personalized workbook to support patients living with achalasia in managing their eating behaviors, particularly in social settings. This innovative approach offers an interesting strategy for improving the quality of life for individuals with achalasia.

Finally, the central nervous system can also affect the GI system through the brain-gut axis. Du et al. investigated the causal relationship between major depressive disorder and functional dyspepsia. The study demonstrated a positive causal relationship, suggesting that severe depression increases the risk of functional dyspepsia. The authors discuss how gut microbiota, the inflammatory response, and hormones can lead to altered GI secretion, inhibited gastric emptying, and altered intestinal motility in patients with major depressive disorder.

In conclusion, the studies featured in this Research Topic provide significant insights into the complex interplay between the gut and the nervous system. By advancing our understanding of the gut-brain axis and the mechanisms underlying GI autonomic disorders, these studies hold the potential to greatly improve the management of patients with these challenging conditions.
